# Pregnancy Prediction in Single Embryo Transfer Cycles after ICSI Using QPCR: Validation in Oocytes from the Same Cohort

**DOI:** 10.1371/journal.pone.0054226

**Published:** 2013-04-03

**Authors:** Sandra Wathlet, Tom Adriaenssens, Ingrid Segers, Greta Verheyen, Lisbet Van Landuyt, Wim Coucke, Paul Devroey, Johan Smitz

**Affiliations:** 1 Follicle Biology Laboratory, UZ Brussel, Belgium; 2 Centre for Reproductive Medicine, UZ Brussel, Brussels, Belgium; 3 Department of Clinical Biology, Scientific Institute of Public Health, Brussels, Belgium; VU University Medical Center, The Netherlands

## Abstract

Cumulus cell (CC) gene expression is being explored as an additional method to morphological scoring to choose the embryo with the highest chance to pregnancy. In 47 ICSI patients with single embryo transfer (SET), from which individual CC samples had been stored, 12 genes using QPCR were retrospectively analyzed. The CC samples were at the same occasion also used to validate a previously obtained pregnancy prediction model comprising three genes (ephrin-B2 (EFNB2), calcium/calmodulin-dependent protein kinase ID, stanniocalcin 1). Latter validation yielded a correct pregnant/non-pregnant classification in 72% of the samples. Subsequently, 9 new genes were analyzed on the same samples and new prediction models were built. Out of the 12 genes analyzed a combination of the best predictive genes was obtained by stepwise multiple regression. One model retained EFNB2 in combination with glutathione S-transferase alpha 3 and 4, progesterone receptor and glutathione peroxidase 3, resulting in 93% correct predictions when 3 patient and treatment cycle characteristics were included into the model. This large patient group allowed to do an intra-patient analysis for 7 patients, an analysis mimicking the methodology that would ultimately be used in clinical routine. CC related to a SET that did not give pregnancy and CC related to their subsequent frozen/thawed embryos which ended in pregnancy were analyzed. The models obtained in the between-patient analysis were used to rank the oocytes within-patients for their chance to pregnancy and resulted in 86% of correct predictions. In conclusion, prediction models built on selected quantified transcripts in CC might help in the decision making process which is currently only based on subjective embryo morphology scoring. The validity of our current models for routine application still need prospective assessment in a larger and more diverse patient population allowing intra-patient analysis.

## Introduction

Single embryo transfer (SET) is the preferred treatment to limit multiple pregnancies after ART. In order not to compromise the carry home baby rate, the selection of the embryo for transfer in the first cycle becomes even more important. Next to the existing criterion based on morphology, other methods are currently under investigation. The use of quantitative gene expression measurements in cumulus cells (CC), which are in close contact with the oocyte during growth and maturation, seems a promising method [1]. Since the first published study on the subject where CC expression could be related to embryo development [2], several other studies have investigated this possibility and try to relate CC expression to different endpoints. Examples of endpoints investigated are: embryo development [3], [4], [5], [6], [7], [8], aneuploidy stage of the oocyte [9], oocyte nuclear maturity stage [10] and probably the most important from a patient perspective: pregnancy outcome [11], [12], [13], [14], [15]. Confirmation of results between different studies does not seem obvious in the analysis of CC gene expression. In the current literature not many genes were found in common in different studies. For example, hyaluronan synthase 2 (*HAS2*) was higher expressed in good quality embryos compared to low embryo morphology in two studies [2], [4], but could not be related to embryo morphology in two other studies [8], [13]. Divergences can be due to a different experimental design, with different endpoints, but gene expression can be influenced by known factors as the stimulation protocol of the patients [16], [17], [18] or not yet assessed factors such as culture media used in the different IVF laboratories.

In this study, 47 individual cumulus complexes from 47 intra-cytoplasmic sperm injection (ICSI) patients were retrospectively analyzed with quantitative real-time polymerase chain reaction (QPCR). Using the current sample set, a pregnancy prediction model from a previous study [15] was validated for its predictive power. In a next step, in an attempt to search for new genes with a stronger predictive power, new multivariable models were built considering the 3 genes (ephrin-B2 (*EFNB2*), calcium/calmodulin-dependent protein kinase ID (*CAMK1D*), stanniocalcin 1 (*STC1*)) described earlier and 9 novel genes (glutathione reductase (*GSR*), glutathione peroxidase 3 (*GPX3*), glutathione S-transferase alpha 3 and 4 (*GSTA3* and *GSTA4*), transforming growth factor beta 1(*TGFB1*), progesterone receptor (*PGR*), inositol 1,4,5-trisphosphate receptor type 1 (*ITPR1*), solute carrier family 2 (facilitated glucose transporter) member 1 (*SLC2A1*) and thrombospondin 1 (*THBS1*)) (inter-patient analysis) (see Table 1 for an overview of all genes).

**Table 1:** Genes analyzed in cumulus cells for pregnancy prediction.

Gene symbol (name)	General Function	Previously described as oocyte quality marker in human CC	References
***EFNB2*** (ephrin-B2)	B-Class Ephrins are transmembrane proteins possibly involved in luteinization events	Higher in the CC of pregnant ICSI patients	[19](A) [15](B)
***CAMK1D*** (calcium/calmodulin-dependent protein kinase ID	Member of the Ca2+/calmodulin-dependent protein kinase 1 subfamily of serine/threonine kinases	Higher in the CC of pregnant ICSI patients	[20](A) [15](B)
***STC1*** (stanniocalcin 1)	Decreases FSH induced progesterone production in rat granulosa cell cultures	Tended to be lower in pregnant ICSI patients	[21], [22](A) [15](B)
***GSR*** (glutathione reductase)	Cellular antioxidant defense enzyme	Not yet described	[23](A)
***GPX3*** (glutathione peroxidase 3)	Helps in the detoxification of hydrogen peroxide	Negative predictor for early cleavage embryos	[23](A) [7](B)
***GSTA3*** (glutathione S-transferase alpha 3)	Detoxification function next to a function in progesterone production	Not yet described	[23], [24](A)
***GSTA4*** (glutathione S-transferase alpha 4)	Detoxification function	Not yet described	[23](A)
***TGFB1*** (transforming growth factor, beta 1)	Can play a role in cell proliferation and differentiation. It was shown to be related to follicle development in adult mice.	Not yet described	[20], [25], [26] (A)
***PGR*** (progesterone receptor)	Anti-apoptotic effect through the binding of progesterone in cultured human granulosa cells	Lower expressed in good morphology blastocysts. Up- regulated in follicular cells of pregnant patients (array results)	[27](A) [3], [28](B)
***ITPR1*** (inositol 1,4,5-trisphosphate receptor, type 1	Receptor for inositol 1,4,5-triphsopahte, releasing calcium from the endoplasmatic reticulum	Up-regulated in non-early cleavage embryos (array results)	[29](A) [7](B)
***SLC2A1*** (solute carrier family 2 (facilitated glucose transporter), member 1)	Glucose transporter responsible for the facilitated transport of glucose through the plasma membrane of mammalian cells	Not yet described	[30](A)
***THBS1*** (thrombospondin 1)	Can mediate cell-cell and cell-matrix interactions. Can activate TGFB1	Not yet described	[31], [32](A)

(A) Refers to information from the ‘General Function’ column; (B) Refers to the information from the ‘Previously described as oocyte quality marker in human CC’ column; CC: cumulus cells; FSH: Follicle stimulating hormone; ICSI: intra-cytoplasmic sperm injection.

Our patient sample set allowed for an analysis never reported before in literature: CC from oocytes that did not result in pregnancy in the fresh transfer cycle and the CC from their sibling oocytes that resulted in pregnancy after a frozen embryo transfer (FRET) cycle were analyzed (intra-patient analysis). To our knowledge this is the first study to compare pregnant and non-pregnant CC from the same retrieval cycle in a SET setting as would be done in a final clinical application.

## Materials and Methods

### Patient population

This study was approved by the Ethical Committee of the UZBrussel and the patients written consent was obtained. Forty seven ICSI patients were selected based on the embryo transfer policy (single embryo transfer) and ovarian stimulation protocol prescribed: GnRH antagonist in combination with recombinant follicle stimulating hormone (FSH) (Gonal-f, Merck-Serono, Geneva, Switzerland; *n* = 4 or Puregon, MSD, Oss, The Netherlands; *n* = 43). Causes of infertility were: male factor only (n = 19), female factor only (ovulation disorder n = 3 and tubal infertility n = 2), combination male and female factor (OAT and endometriosis n = 3) and idiopathic (n = 20). Twenty patients had single embryo transfer on day 3 of culture, from these 10 became pregnant. Twenty seven patients had transfer on day 5, from these 9 became pregnant. The day of transfer was decided by the consulting doctor before oocyte retrieval took place taking into account the age of the patient or by the embryologist according to the number of good embryos available. All pregnancies resulted in live births.

### Collection of human cumulus cells and embryo culture

Vaginal ultrasound was used to monitor follicular development. The endocrine profile was monitored by analysis of serum 17β-estradiol (E2), progesterone, FSH and LH by electrochemiluminescence on a COBAS 6001 immunoanalyser (Roche, Roche Diagnostics, Mannheim, Germany) using validated assays with respectively sensitivities of 5 ng/l, 0.03 µg/l, <0.1 IU/l, 0.1 IU/l and total imprecisions (%CV) of respectively <6, <7, <6 and <6. Final follicular maturation was induced with a dose of 10 000 IU hCG when at least three follicles of 17 mm in diameter were observed by transvaginal ultrasound. Oocyte retrieval was done 36 h later. CC collection was done as described in [14]. Briefly, individual oocyte denudation was performed in 40 µl droplets of HTF-SSS containing 80 IU/ml Cumulase (MediCult, Lyon, France) for not longer than 30 s and washed sequentially in droplets without enzyme. At any time, oocytes were handled individually from this point onwards in order to allow retrospective analysis of the CC per oocyte. After denudation, the CC were plunged directly in liquid nitrogen. ICSI was performed as described previously [33] and embryos were cultured in sequential media of SAGE (CooperSurgical, Leisegang Medical, Berlin). Embryos were vitrified on day 3 or day 5/6 of embryo culture as was described earlier [34] and used in a subsequent FRET cycle. The day 3 embryos were warmed on cycle day 3, cultured overnight and transferred as a day 4 embryo on cycle day 4 ( = synchronized transfer). The day 5 (and day 6) blastocysts were warmed in cycle day 5 (or day 6) in the morning and transferred on the same day.

**Figure 1:**
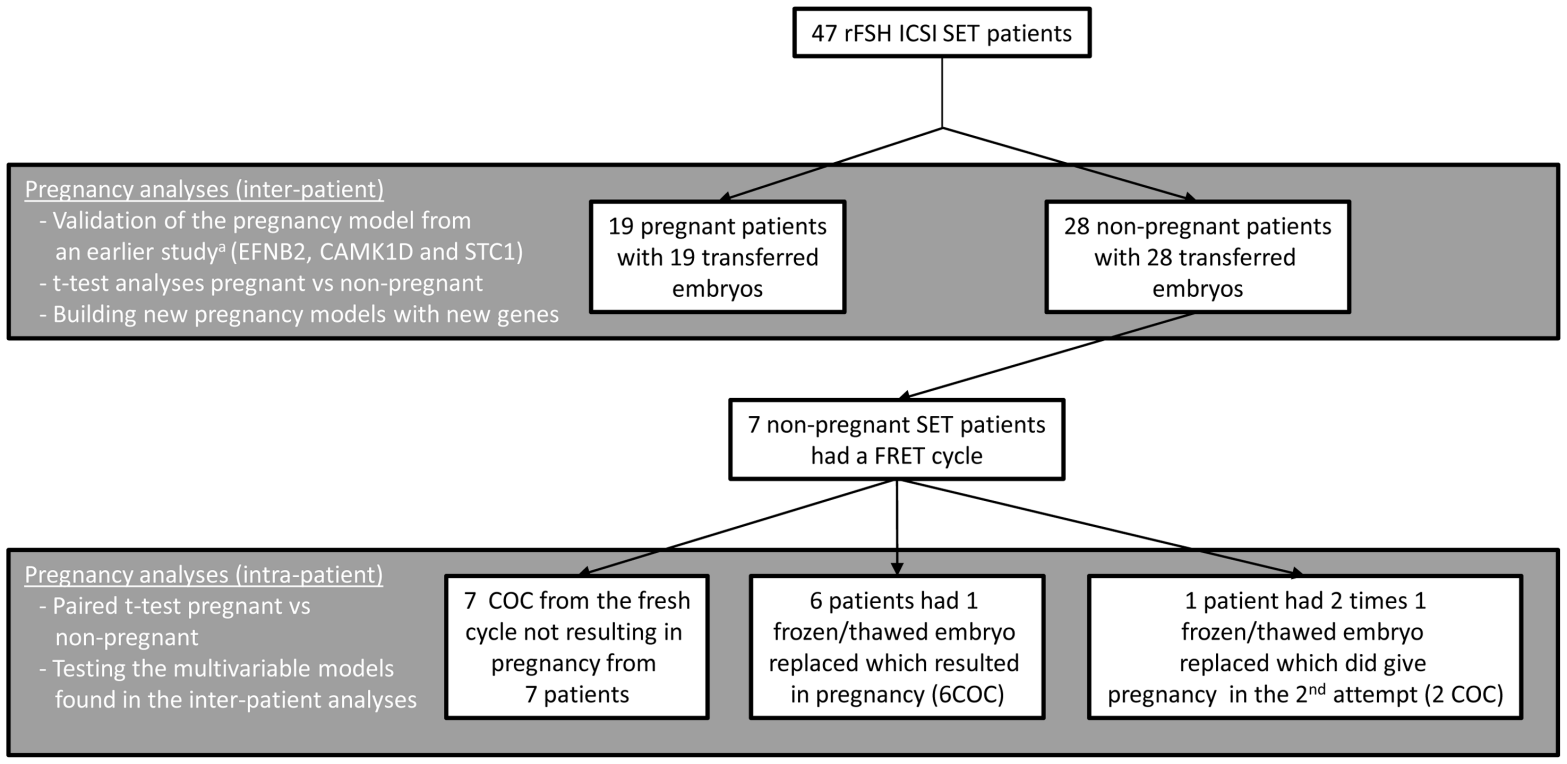
Overview of the samples used in this study. This figure represents the distribution of the samples used for the different analyses performed in this study. The grey background fields delimit the samples that were used for the specific analyses which are marked on the left side of the field. SET: single embryo transfer; FRET: frozen embryo transfer cycle; rFSH: recombinant Follicle Stimulating Hormone.^a^:[15].

For all 47 patients, only the CC related to those oocytes resulting in embryos selected for transfer were analyzed exception made for 7 of the 28 non-pregnant patients, 1 extra CC sample (except for 1 patient, 2 CC samples) related to a vitrified embryo giving pregnancy after a frozen single-embryo transfer cycle, was analyzed (8 extra CC in total from 7 patients). Figure 1 shows the different samples used for each analysis.

### Gene selection

This study is the 3^rd^ one in a row to evaluate the predictive value of CC gene expression for oocyte quality using QPCR. Over the 3 studies, we followed a precise strategy to choose which genes to analyze regarding to oocyte quality in ICSI patients. The first study identified 4 top genes, 2 predictive for embryo morphology (inositol-trisphosphate 3-kinase A (*ITPKA*) and transient receptor potential cation channel, subfamily M, member 7 (*TRPM7*)) and 2 for pregnancy outcome (syndecan 4 (*SDC4*) and versican (*VCAN*)). It was chosen to include those 4 genes in the next study [15]. *ITPKA* and *TRPM7* were again related to embryo development, but *SDC4* and *VCAN* were not retained this time in the pregnancy models and were replaced by *EFNB2*, *CAMK1D* and *STC1* when using stepwise multiple regression analysis. As several studies showed no relation between the genes predictive for embryo development and pregnancy outcome [13], [15], it was decided to repeat only the 3 pregnancy related genes in this third study. Our hypothesis is that by this ‘cascade’ testing strategy, the strongest pregnancy predictive genes may be filtered out through the consecutive studies (Table 2). The other 9 genes analyzed in this study were: *GSTA3, GSTA4, GPX3, GSR, ITPR1, SLC2A1, THBS1, TGFB1* and *PGR* (Table 1). The 9 new genes were chosen from own unpublished array data comparing CCs related to pregnancy versus CCs related to no pregnancy.

**Table 2:** Strategy over 3 studies to obtain the strongest quality related genes on stored cumulus cells from ICSI patients.

	Wathlet et al. 2011	Wathlet et al. 2012	Current study
Genes tested with QPCR	*SDC4, VCAN, ITPKA, TRPM7, PTGS2,* *GREM1, CALM2, ALCAM*	***SDC4, VCAN, ITPKA, TRPM7*** *, CAMK1D,* *EFNB2, STC1, STC2, CYP11A1, HSD3B1, PTHLH*	***CAMK1D, EFNB2, STC1*** *, GSTA4, GSTA3, GSR, GPX3, PGR, THBS1, SLC2A1, ITPR1, TGFB1*
Stimulation protocol	antagonist rFSH (25 patients) agonist HP-hMG (20 patients)	Antagonist rFSH (33 patients)	Antagonist rFSH (47 patients)
Embryo culture medium	BlastAssist System (Medicult)	Vitrolife G7 (Vitrolife)	SAGE (CooperSurgical)
End points	Embryo morphology (75 CC for rFSH and 67 CC for HP-hMG from 2×10 patients). Clinical pregnancy (42 patients of both stimulation protocols of which 19 pregnant = 19 COC)	Embryo morphology (99 CC). Biochemical and live birth pregnancy (16 pregnant, 17 non-pregnant = 33 COC)	Live birth pregnancy inter-patient analysis (19 live birth, 28 non-pregnant = 47 COC). Pregnancy intra-patient (7 patients with 2 or 3 CC from the same retrieval cycle)
Best genes retained for next study	Pregnancy prediction: *SDC4* and *VCAN.* Embryo morphology prediction: *ITPKA* and *TRPM7*	Pregnancy prediction: *CAMK1D, EFNB2* and *STC1*	To be determined in the current study

The gene expression of cumulus cells (CC) related to different embryo morphology or pregnancy outcome of the corresponding oocytes in ICSI was assessed for three gene panels on three different sample sets. The genes found most predictive in each sample set were tested in the subsequent independent patient samples set. Genes marked in bold were retained as best predictive genes from the previous study. MII: metaphase II oocytes; COC: cumulus oophorus complex; rFSH: recombinant Follicle Stimulating Hormone; HP-hMG: Highly Purified human Menopausal Gonadotropin.

### RNA extraction and cDNA synthesis

Total RNA was extracted as described earlier [17] using the RNeasy Micro Kit (Qiagen, Westburg, Leusden, The Netherlands) including a DNase step and addition of 5 ng/µl poly(dA) (Roche Applied Science, Mannheim, Germany) prior to extraction. Extraction was followed by a second DNase treatment (RQ1 RNase-Free DNase, Promega, Leiden, The Netherlands).

Reverse transcriptase (RT) was done as previously described [17] with the iScript cDNA Synthesis Kit (Bio-Rad Laboratories, Ghent, Belgium). Negative controls were generated by omitting the enzyme or the RNA in the RT reaction.

### Real-time PCR

Primer sequences for *CAMK1D, STC1* and *EFNB2* are listed in Wathlet et al 2012. Primers for *GPX3, GSTA3, GSTA4, PGR, THB1, ITPRA, SLC2A1, GSR* and *TGFb1* can be found in Table S1. Both beta-2-microglobulin (*B2M*) and ubiquitin C (*UBC*) were validated and used before as normalization factor [14]. Cycling conditions, negative controls, standard curves and normalization (with *B2M* and *UBC*) are as described earlier [14], but all PCR reactions were adapted to 10 µl reactions. All values mentioned hereafter are the normalized values to the mean of both *B2M* and *UBC* for each sample.

### Statistics

#### Inter-patient analysis

In a first analysis, a two-tailed t-test (GraphPad Prism version 4.01 for Windows, GraphPad Software, San Diego California USA) was used to compare cumulus complexes of oocytes resulting in pregnancy or not (19 live birth, 28 non-pregnant). All data were LOG transformed to obtain normal distribution and only *P*-values <0.0042 were considered significant after Bonferroni correction.

In a second analysis, a model was built using stepwise multiple regression analysis as described earlier [14]. Briefly, a linear regression model, with an equation as output ‘y = a + bx + cz + ds +et + fu + gv + hw’, was built with as response variables (x, z, s, t, u, v, w) gene expression and/or patient and cycle characteristics (all are listed in Table 3). ‘b–h’ are the respective indexes of the included variables and ‘a’ is the intercept of the equation. A variable was added to the model if the type III *P*-value of the variable was <0.3 and if the *P*-value of the model was improved. At the end of the model a backwards regression step was performed to exclude redundant variables. Four different models were built to predict pregnancy. In two models only gene expression values were allowed (first the model was restricted to 3 genes, next all genes were allowed into the models as long as they improved the *P*-value of the model). In two other models, the need for correction by patient and cycle characteristics was assessed by allowing all patient and cycle characteristics to the models only composed of genes, when those extra variables could improve the model. By introducing those extra factors, possible inter-patient variability on gene expression could be leveled out and increase the differences related to oocyte quality.

**Table 3:** Patient and cycle characteristics.

Pregnant					Non-pregnant			
Variable	Unit	average	SD	n	average	SD	n	t-test
**Age**	Year	30	4	19	31	5	28	ns
**BMI**	kg/m2	23	4	17	23	4	25	ns
**Days of stimulation**	#	9	2	19	8	1	28	ns
**Gonadotrophine dose**	U/day	167	34	19	169	36	28	ns
**FSH^a^**	U/l	11	3	16	12	4	26	ns
**LH^a^**	U/l	1.88	1.46	10	1.18	0.86	24	ns
**Relative E2**	ng/l	150	98	16	162	87	26	ns
**Progesterone^a^**	µg/l	0.79	0.26	16	0.77	0.42	26	ns
**COC retrieved at pick up**	#	10	5	19	9	5	28	ns
**Ovarian Response**	#	6	3	19	6	3	28	ns
**Oocyte Maturity**	%	89	10	19	80	15	28	ns
**2PN**	%	82	15	19	87	16	28	ns
**≥7cell day3**	%	74	25	19	72	29	28	ns
**Low Fragmentation**	%	65	30	19	73	29	28	ns
**Good Quality Embryos**	%	58	17	19	53	29	28	ns

COC: cumulus oocyte complex; Relative E2: E2/COC retrieved; Ovarian Response: (COC retrieved/Gonadotrophine dose) x 100; Oocyte Maturity: proportion of MII/COC retrieved; 2PN: proportion of 2PN/intact oocytes after ICSI; ≥7cell day3  =  proportion of embryos with at least 7 cells on day3/2PN; Low Fragmentation: proportion of embryos with <10% fragmentation on day3/2PN; Good Quality Embryos: proportion of embryos on day 3 with <10% fragmentation and at least 7 cells/2PN; ^a^ Serum values as measured on day of hCG; ns: *P*>0.05; SD =  standard deviation.

For all models, accuracy ((True positive + true negative)/(true positive + false positive + false negative + true negative)) and positive and negative predictive values (PPV =  True positive/(true positive + false negative) and NPV  =  True negative/(true negative + false positive) were calculated. The power of all models was represented by Receiver Operating Characteristic (ROC) and the area under curve (AUC) was calculated.

#### Intra-patient analysis

Finally, the study allowed comparing 2 (or 3) oocytes originating from one oocyte retrieval cycle with known pregnancy outcome per oocyte, as all embryos were transferred individually in consecutive cycles. For this purpose, from 7 of the 28 patients that were not pregnant in the fresh cycle, the CC of the embryos that were replaced in a subsequent frozen single embryo transfer cycle resulting in pregnancy were compared to those transferred in the fresh cycle. For one patient, two consecutive frozen embryo replacement cycles were analyzed as the first embryo did not end in a pregnancy. Seven genes were chosen for this analysis based on their presence in one of the above mentioned models or their *P*-value of addition, when first added to a pregnancy model. A paired t-test was performed for each gene and the chance to pregnancy was calculated with the earlier defined models from the inter-patient analysis, containing only genes and built on the 47 CC samples (19 live birth), but excluding the 8 CC samples from the frozen cycles.

## Results

### Patient population

No statistical differences were found between the patient and cycle characteristics of the pregnant and the non-pregnant groups (Table 3). Mean age and BMI for both groups of patients was low but comparable. Progesterone levels were low in both groups. Mean cycle number attempt was not different in both groups: respectively in the pregnant and non-pregnant group 12 and 17 patients underwent their first cycle, 6 and 10 their second cycle and 1 for both groups their third cycle. Percentages of oocyte maturity and fertilization were normal in both groups, and the percentage of good quality embryos on day 3 was more than 50%. Embryo quality score on moment of transfer was not different for both groups. Nineteen fresh SET cycles resulted in live birth.

### Pregnancy prediction

#### Validation of predictive genes (CAMK1D, EFNB2 and STC1) using a model built on a previous sample set

A model predicting pregnancy composed of 3 genes, considered in a previous study [15], was tested in the current, independent patient series. The gene expression values for *CAMK1D, EFNB2* and *STC1* of the CC of the 47 patients were introduced in the equation obtained before and gave a value predicting the chance to pregnancy for each of the 47 oocytes. The obtained PPV and NPV calculated with the 47 samples of this study were 62% and 86%, with an accuracy of 72%.

#### Inter-patient analysis: t-test of all 12 genes in the current sample set

**Figure 2:**
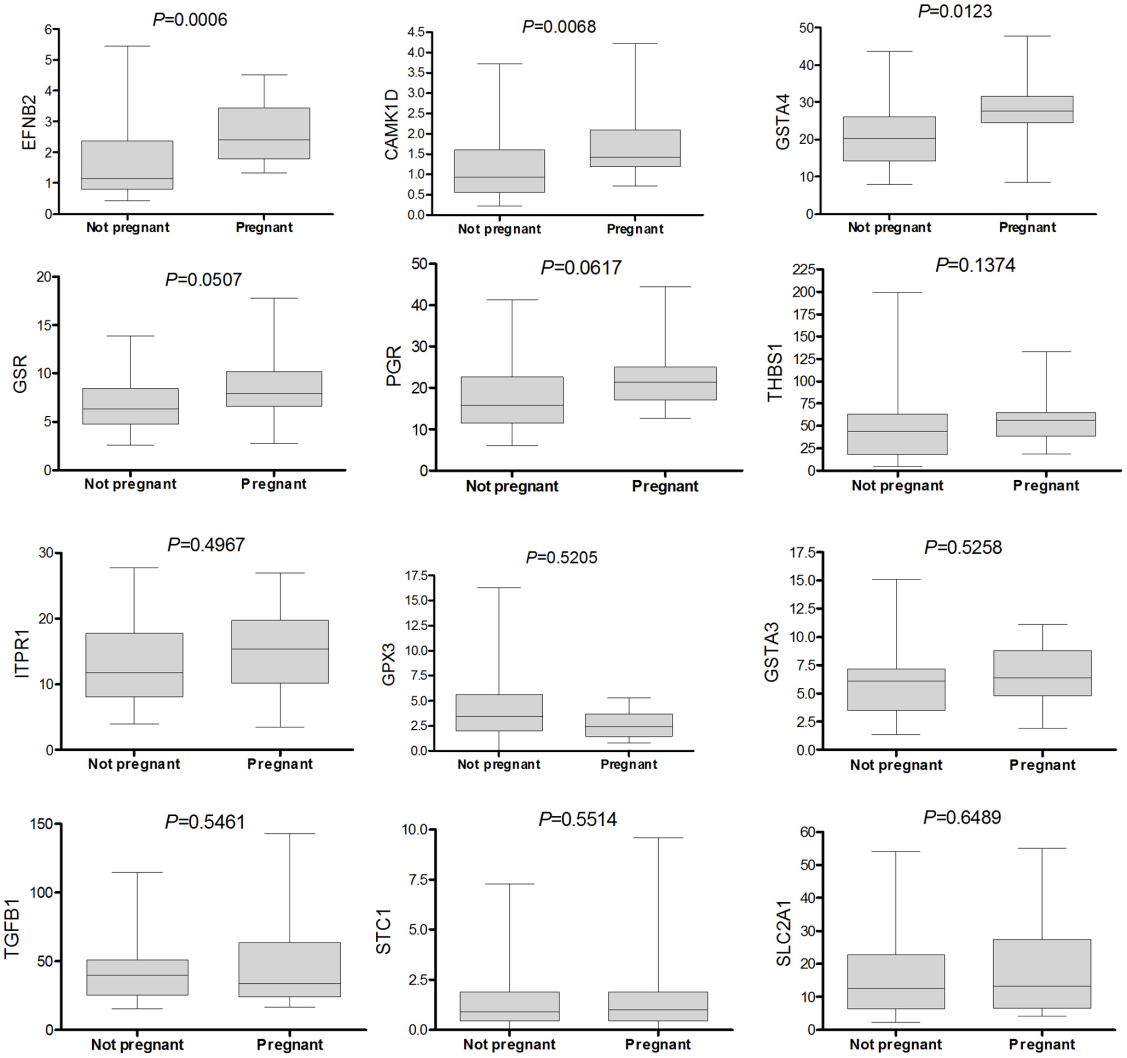
t-test of normalized gene expression values of non-pregnant versus live birth related cumulus cell samples. The graphs represent the differences in gene expression between the cumulus cell samples associated to an oocyte that after in vitro fertilization treatment resulted in a live birth (n = 19) or not (n = 28). Normalization was done to the mean of *B2M* and *UBC*. Only *P*-values <0.0042 were considered significant after Bonferroni correction. The total range of expressions found is depicted by the boxes and whiskers respectively representing the two inner and the two outer quartiles with centrally the median.

The cumulus complexes of 28 oocytes not resulting in pregnancy were compared to 19 CC of oocytes that resulted in a live birth. Only *EFNB2* was statistically higher in the pregnant group. *CAMK1D, GSTA4* and *GSR* only showed a trend of higher expression in the pregnant group (respective *P*-values: 0.0068, 0.0123 and 0.0507). Graphs for all genes can be found in Figure 2.

#### Inter-patient analysis: Stepwise multiple regression analysis

In a first step to build a pregnancy model, the *P*-value of addition when added as first variable was calculated for all genes and can be found in Table 4.

**Table 4:** *P*-value of addition for the different genes tested.

Variable	*P*-value of addition	Variable	*P*-value of addition
*EFNB2*	0.01	*STC1*	0.38
*GSTA4*	0.01	*TGFB1*	0.43
*GPX3*	0.04	*ITPR1*	0.50
*CAMKID*	0.05	*SLC2A1*	0.61
*GSR*	0.06	*THBS1*	0.68
*PGR*	0.16	*GSTA3*	0.75

The *P*-value of addition is obtained when each gene is inserted as first variable in a pregnancy model. The genes are ordered with increasing *P*-value.

First, the model was restricted to the inclusion of 3 of the 12 genes (Model 1). *EFNB2, GSTA4* and *PGR* were retained and gave a model with a *P*-value of 0.0015, a PPV of 68%, a NPV of 79% an accuracy of 73% and an AUC of 0.82. When trying to improve this model by also allowing patient and cycle characteristics (from Table 3), no improvement on the previous model was found (Model 1 bis).

**Figure 3:**
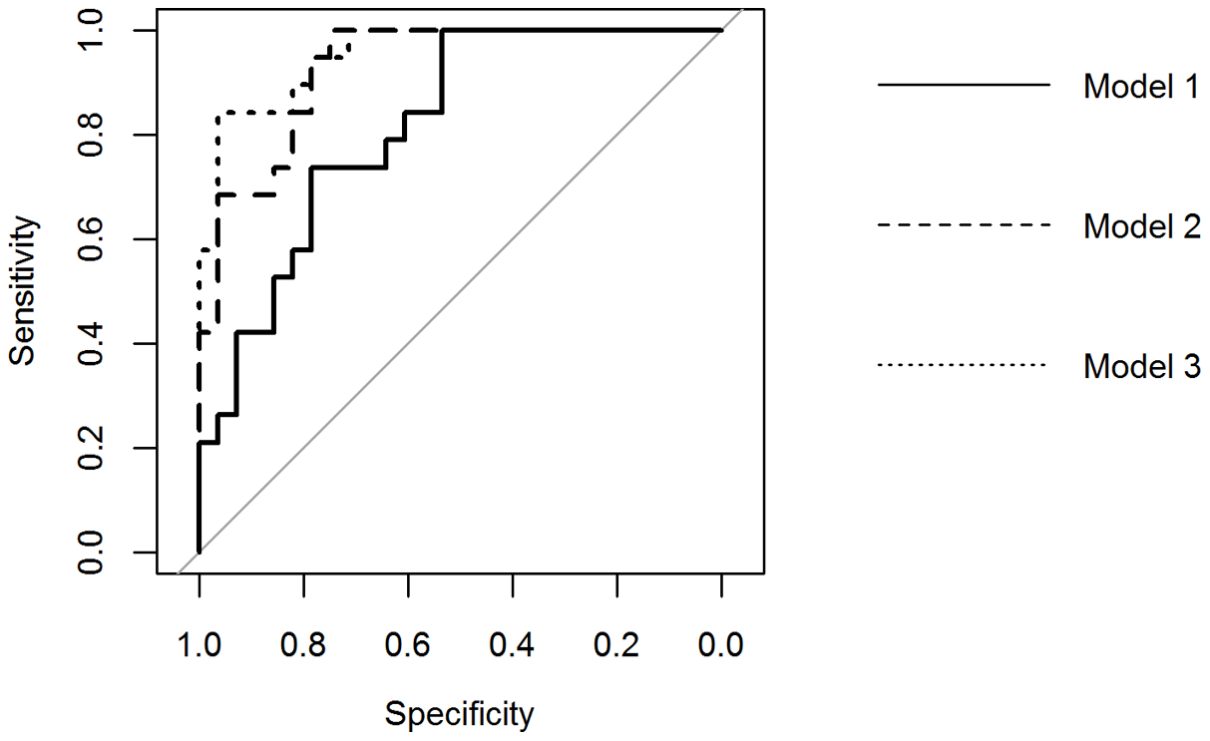
Receiver operating characteristic (ROC) curve of the 3 pregnancy models. Multivariable models were built to predict the chance to pregnancy including the gene expression levels measured in cumulus cell samples associated to an oocyte that after in vitro fertilization treatment resulted in a live birth or not. For Model 3 patient and cycle characteristics were also included (from Table 3). Model 1 was limited to 3 genes and is composed of *EFNB2, PGR* and *GSTA4*. In Model 2 all genes were allowed into the model as long as they could improve the model. Five genes were retained for Model 2: *EFNB2, PGR, GSTA4, GSTA3, GPX3*. To try to improve Model 2, in Model 3 patient and cycle characteristics were allowed into the model if they could improve the *P*-value of the model: *EFNB2, PGR, GSTA4, GSTA3, GPX3*, age, Relative E2, and number of days of ovarian stimulation. The respective areas under the curve are 0.82, 0.93 and 0.95. Relative E2: E2 value measured on day of hCG over the number of cumulus oophorus complexes.

In a next step, more than 3 genes were allowed into the model if improving the *P*-value (Model 2). Five of the 12 genes were retained in this model (i.e. *EFNB2, GSTA4, PGR, GPX3* and *GSTA3*) which yielded a *P*-value <0.0001 with a PPV of 78%, a NPV of 83%, an accuracy of 81% and an AUC of 0.93. Adding patient and cycle characteristics improved the model (Model 3). The retained parameters were: days of stimulation, relative E2 and age. The PPV, NPV and accuracy of the extended model all increased to 93% and the AUC to 0.95 (Table 5). Full mathematical models can be found in Table 6. ROC curves are shown in Figure 3.

**Table 5:** Schematic overview of the multivariable models for live birth prediction.

	Total # of patients	# of pregnant patients	GPX3	GSTA3	GSTA4	PGR	EFNB2	AGE	Rel E2	# Days stim	P model	PPV (%)	NPV (%)	Accuracy (%)	AUC
Model 1 (3 genes)	47	19			x^a^	x	x^a^				0.0015	68	79	73	0.82
Model 1 bis (Model 1 + patient and cycle parameters)	*cycle and patient parameters could not improve the first model*														
Model 2 (unlimited # of genes)	47	19	x^a^	x^a^	x^a^	x	x				0	78	83	81	0.93
Model 3 (Model 2 + patient and cycle parameters)	42*	16	x^a^	x^a^	x^a^	x^a^	x^a^	x	x	x	0	93	93	93	0.95

Only genes and factors that were at least retained once are listed. In Model 1 a maximum of three genes were retained to finalize the model. In Model 2 an unlimited number of genes were allowed into the model, if they could improve the *P*-value of the model. To try to improve Model 1 and Model 2 patient and cycle characteristics from Table 3 were allowed into the model. Only Model 2 could be improved and resulted in Model 3. x: factor significantly improving the model; #: number; ‘unlimited’ refers to the fact that all 12 genes were allowed into the model if they could improve the model; stim: ovarian stimulation; PPV: positive predictive value; NPV: negative predictive value; AUC: Area under the curve; ^a^: Final type-III *P*-value <0.01 in the model;* only 42 patients were included to build this model as E2 values on day of hCG were missing for 5 patients.

**Table 6:** Mathematical models for pregnancy prediction.

Model 1 (max 3 genes) =	−2.25846+0.79256× *EFNB2*+0.09491× *GSTA4* –0.09632× *PGR*
Model 1bis (max 3 genes + parameters Table 3) =	*cycle and patient parameters could not improve the first model*
Model 2 (unlimited # of genes) =	−1.02049+0.63484× *EFNB2*+0.27346× *GSTA4* –0.10864× *PGR* – 0.43395× *GPX3* –0.51067× *GSTA3*
Model 3 (unlimited # of genes + parameters Table 3) =	−11.26732 + 1.3462× *EFNB2*+0.45884× *GSTA4* –0.2423× *PGR* –0.65786× *GPX3* –0.85875× *GSTA3* +0.49709× days of stimulation + 0.0092× Rel E2 +0.13864× age

Full mathematical models used to predict pregnancy outcome using cumulus cell gene expression values and patient and cycle characteristics. #: number.

#### Intra-patient pregnancy prediction

For seven patients, cryostored CC samples related to cryopreserved embryos which had led to a clinical pregnancy after transfer in a subsequent transfer cycle were analyzed. This material was used to analyze the genes present in the above obtained multivariable models (Table 5) and/or the 5 genes with the smallest *P*-value of addition (Table 4) i.e. *CAMK1D, EFNB2, GPX3, GSR, GSAT4, GSTA3* and *PGR*. As a first explorative method a paired t-test for those seven genes was performed and can be found in Figure S1. Five genes had an upwards trend in the CC of an oocyte resulting in pregnancy (*P*-value <0.05; only *P*-values <0.07 are significant after Bonferroni correction for multiple comparisons): *EFNB2* (only significant difference between pregnant and non-pregnant), *CAMK1D, GSR* and *PGR*. *GSTA4, GPX3* and *GSTA3* had all *P*-values >0.05. Fold changes between the CC from the same patient were calculated and an average per gene was made (from 1.1 to 2.5) (Table 7). For the five genes with a *P*-value <0.1 in the paired t-test, the percentage of correctly estimated CC was 71% to 88%, based on the level expected (e.g. higher expression is expected in the pregnant compared to the non-pregnant related CC) from the paired t-test. In a next step the predictive power of the multivariable models (the 3 gene model ( = Model 1) and the 5 gene model ( = Model 2)) obtained in the inter-patient analysis (47 CC samples) was used to rank the CC of each patient (Table 5) for their chance to pregnancy. As all patient and cycle characteristics are identical for oocytes within a single retrieval cycle it makes obviously no sense to use models containing those variables. All CC, except for patient 4, were correctly ranked (Table 7) for their chance to pregnancy.

**Table 7:** Comparison of gene expression levels of fresh cycles not resulting in pregnancy to frozen transfer cycles resulting in pregnancy (intra-patient analysis).

				Single gene analysis							Multivariable	
											Model 1	Model 2
	Fresh/FRET	Outcome		*EFNB2*	*CAMKID*	*GSR*	*PGR*	*GSTA4*	*GSTA3*	*GPX3*	Ranking	Ranking
Patient 1	Fresh	not pregnant	ratio	3.4	3.5	1.8	2.1	1.4	5.4	0.7	2	2
	FRET	clinical pregnancy									1	1
Patient 2	Fresh	not pregnant	ratio	4.9	4.9	2.3	4.0	2.6	0.0	2.9	2	2
	FRET	clinical pregnancy									1	1
Patient 3	Fresh	not pregnant	ratio	1.8	1.8	1.6	1.8	1.4	0.0	0.6	2	2
	FRET	clinical pregnancy									1	1
Patient 4	Fresh	not pregnant	ratio	1.6	1.8	1.4	3.6	0.7^ a^	1.1	1.0	1^ b^	1^ b^
	FRET	clinical pregnancy									2^ b^	2^ b^
Patient 5	Fresh	not pregnant	ratio	4.1	3.2	4.1	1.5	1.2	1.2	1.0	2	2
	FRET	Live birth									1	1
Patient 6	Fresh	not pregnant	ratio	1.4	1.3	1.3	1.0^ a^	1.3	1.2	0.8	3	3
	FRET	not pregnant	ratio	1.0^ a^	0.6^ a^	0.6^ a^	0.7^ a^	0.9^ a^	0.8	0.6	2	2
	FRET	clinical pregnancy									1	1
Patient 7	Fresh	not pregnant	ratio	2.3	2.8	2.9	2.8	3.1	1.6	1.3	2	2
	FRET	Live birth									1	1
Based on the paired t-test expected higher in:				pregnant	pregnant	pregnant	pregnant	pregnant	nlr	nlr		
Average:				2.5	2.5	2.0	2.0	1.6	1.6	1.1		
Min:				1.0	0.6	0.6	0.6	0.7	0.7	0.6		
Max:				4.9	4.9	4.1	4.1	3.1	3.1	2.9		
% corrected predictions based on expression level:				86	86	86	86	71	na	na	86%	86%
*P*-value paired t-test:				0.006	0.026	0.037	0.033	0.074	ns	ns		

This Table gives an overview of the intra-patient analysis. Each line represents one cumulus complex. Per patient 2 or 3 cumulus complexes from 1 retrieval cycle were analyzed. Ratios of gene expression levels are always pregnant over non-pregnant. For patient 6, the cumulus complex of the pregnant FRET cycle was compared to the cumulus complexes of the fresh and the FRET non-pregnant cycle. Model 1 and 2 are respectively the models built up with 3 and 5 genes from Table 5. Ranking was obtained by inserting the expression values in the mathematical models from Table 6. Rank number ‘1’ is the oocyte with the highest chance to achieve pregnancy. ns: not significant; na: not applicable; nlr: no linear relation. ^a^: the expression value is not higher or lower between pregnant and non-pregnant as expected based on the earlier results. ^b^: the ranking is not correct using the multivariable models. FRET: frozen embryo transfer cycle.

## Discussion

The expression of 12 genes in the CC and their capacity to predict the pregnancy potential of the oocyte they surrounded were analyzed in the present study. Three genes (*CAMK1D, EFNB2* and *STC1*) were included from a previous study [15], where they were coming out as the most predictive ones for pregnancy prediction. *EFNB2* was also significantly up-regulated in the current study in the CC from the oocytes that gave pregnancy and *CAMK1D* showed the same trend (*P* = 0.0068). The model composed of genes only from the previous patient dataset, using *CAMK1D, EFNB2* and *STC1*, was validated in this independent patient group and yielded a similar overall performance of the model, with accuracies of 72% in the current study and 79% in the previous study.

Besides the *EFNB2* and *CAMK1D* coming from our previous study, two newly studied genes, *GSTA4* and *GSR*, also showed an up-regulated trend in the CC of the pregnant group.

A multivariable approach tested whether it would be possible to predict live birth by using only 3 of the 12 tested genes in the current sample set (inter-patient analysis). The 3 most predictive genes were *GSTA4*, *PGR* and *EFNB2* and resulted in a similar accuracy as the previous model with *EFNB2, CAMK1D* and *STC1* (73% versus 72%). Using three genes, none of the patient or cycle characteristics could improve the model, suggesting that the expression of the three genes was minimally influenced by patient and cycle factors. The live birth model could be improved by including two more genes (*GPX3* and *GSTA3*), which increased the accuracy up to 81% and resulted in an AUC of 0.93. This 5 gene model was improved with three patient and cycle characteristics (age, relative E2 and days of stimulation) and resulted in an optimized model with a PPV, NPV and accuracy of 93%, but similar AUC as the model only containing genes. Ideally, pregnancy prediction based on gene expression should be possible with a limited set of genes to reduce analysis time and cost. Of the 12 genes tested in this patient population, the 2 most recurrent genes are *GSTA4* and *EFNB2* (see Table 5). These 2 genes are present in all 3 pregnancy models and have respectively in 3 out of 3 and in 2 out of 3 predictive models a type-III *P*-value <0.01. The models also showed that the gene expression values are always more important than the patient and cycle characteristics, as the type-III *P*-values in the models are only significant for the genes and not for the patient and cycle characteristics. Furthermore, the AUC results are comparable between the model containing only genes and the model combining genes and patient and cycle characteristics.

For 7 patients, 2 (or 3) CC from oocytes giving embryos with a good morphology and consecutively transferred, were analyzed (intra-patient analysis). For all oocytes, pregnancy outcome was known as all embryo transfers were done in subsequent SET cycles. Having assessed the expression of 7 genes (*EFNB2, CAMK1D, GSR, PGR, GSTA4, GSTA3* and *GPX3*), it was easy to estimate, based on single genes or on a combination of them in a model, which oocyte of the two considered ones would have the highest chance to pregnancy. The best predictions, when looking at each gene individually, were found for *CAMK1D, GSR, EFNB2, PGR* and *GSTA4* with 71% to 86% correct estimations. Combining 3 or 5 genes, according to the earlier models, 6 of the 7 patients had the cumulus complexes correctly predicting the chance to pregnancy. This prediction was the same when using the 3 or 5 gene model. Surprisingly, the multivariable model failed on a different patient than the single gene analysis. The multivariable approach seems stronger than the individual genes as all genes wrongly predicted the pregnancy outcome for the second comparison of patient 6, but the multivariable model predicted successfully, especially as the CC of the oocytes that resulted in pregnancy were not considered for building the predictive model. The failed prediction of the multivariable approach for patient 4, where the individual genes were correct in 4 out of 5, could be due to other factors (e.g. the endometrium status at the moment of transfer). Although this analysis is still limited in patient numbers, these results are encouraging. This is the first study that confirms that some genes predicting pregnancy between patients might also be capable of ranking the quality of oocytes within patients using a multivariable approach and providing a chance to pregnancy for each oocyte. CC gene expression analysis might become a valuable tool in the ART lab but does obviously not take into account the eventual influence of poor sperm quality and out-of-phase endometrium.

This study could confirm the use of earlier found predictive genes in a new patient population, with the same stimulation protocol, but with different culture media. This data suggests that *EFNB2* and *CAMK1D*, the only genes that we analyzed in two studies using two different culture media, were not affected by culture media. To test the validity of the current models, a future analyze should involve patients with different stimulation protocols and different culture media. Reasons why the specific genes of this study are important for pregnancy prediction remains speculative. As mentioned previously [15], *CAMK1D* may, among other pathways, be related to steroidogenesis by its strong correlation with steroid related genes (*CYP11A1, STC2* and *HSD3B1*). In this data set *CAMK1D* also strongly correlated with a steroid related gene i.e. *PGR* next to *EFNB2* and *GSTA4* (all *P*<0.0001 with Pearson correlation analysis) still leaving the possibilities open for more than one pathway to which *CAMK1D* could be associated with. The presence of *PGR* in the pregnancy models and its predictive power within patients might indicate that steroid-related genes could be helpful in pregnancy prediction. *PGR* has been described before, but no difference between pregnant and non-pregnant could be confirmed with quantitative PCR [28]. *GSTA3* that was present in some of the pregnancy models also has a link with progesterone production [24] reiterating the importance of the steroidogenesis pathway. The function of *EFNB2* in the ovary is not yet known, but B-class ephrins were proposed to be related to the luteinization process [19] and the ephrin B2 receptor was found differently expressed between CC from normal oocytes compared to aneuploid oocytes [9]. Out of the other genes tested, only members of the glutathione family were retained in the models or were significant in the t-test. Glutathione enzymes are important for detoxification actions (of free radicals) in the cells through the use of glutathione. Hypoxia leads to the formation of reactive oxygen species (ROS) which can cause lipid peroxidation, enzyme inactivation and cell damage, resulting in apoptosis [35] not only in CC, but also in the oocyte [36]. Oxidative stress has already been reported by other groups as a possible target to assess oocyte quality [7], [37]. One reason that some transcripts of those pathways are higher in the CC from oocytes giving pregnancy might be that those oocytes are better protected against a stressful environment if needed. Before knowing what are exact role and importance of the considered genes in this study, more experiments need to be performed using animal models to be able to access the antral growth and periovulatory period since in human only 1 time point (i.e. 36h after hCG) is available for analysis.

### Conclusion

By testing presumably important genes batch wise in three consecutive studies on different patient groups for which cumulus complexes were frozen individually per oocyte, we retained the most predictive genes for pregnancy and opposed these every time to new candidate genes. This ‘cascade’ strategy attempted to increase the power of pregnancy prediction using CC gene expression as quality marker for oocytes in ART. The strategy proved effective as the model with *EFNB2*, *CAMK1D* and *STC1* from the second study [15] could be confirmed on an independent patient sample set. In an attempt to further improve prediction models for live birth, models were built (inter-patient analysis), still retaining *EFNB2* together with *PGR* and genes related to the glutathione metabolism. The new models proved to be able to rank oocyte for their potential for pregnancy (intra-patient analysis). The validity of our current models, for routine application, still need prospective assessment in a larger and more diverse patient population allowing intra-patient analysis.

## Supporting Information

Figure S1 **Paired t-test for the intra-patient analysis pregnant versus non-pregnant.** The graphs compare for each patient the gene expression of a cumulus complex that corresponded to an oocyte that did not result in pregnancy to one that resulted in pregnancy in a subsequent single embryo frozen transfer cycle. Per patient the oocytes originate from one retrieval cycle. One patient had 2 consecutive frozen cycles, the first one not resulting in pregnancy. One color represents one patient. The dashed lines show (only in the graphs with a major trend: up or down from non-pregnant to pregnant with *P*<0.1) the pairs not following the major trend. Those pairs are also marked with ‘^a^’ in Table 7.
(PDF)

Table S1 **Primer sequences.**
(DOC)
