# Supplementary figures and images for: Pregnancy Prediction in Single Embryo Transfer Cycles after ICSI Using QPCR: Validation in Oocytes from the Same Cohort
Source: PLoS One. 2013 Apr 3;8(4):e54226. doi: 10.1371/journal.pone.0054226 (PMC3616108; doi:10.1371/journal.pone.0054226)

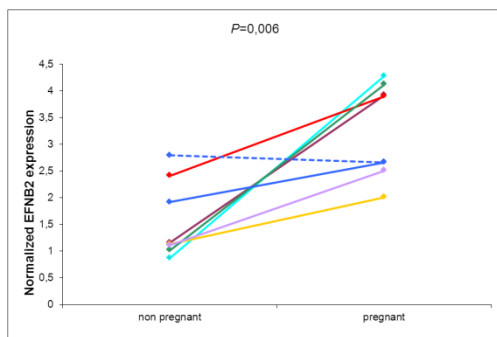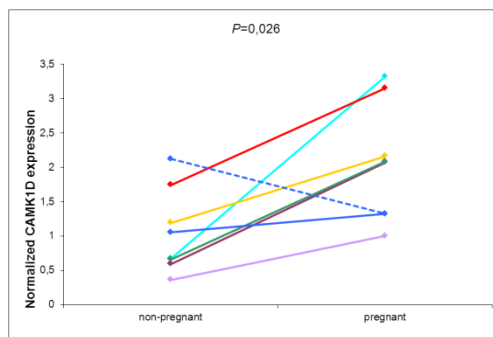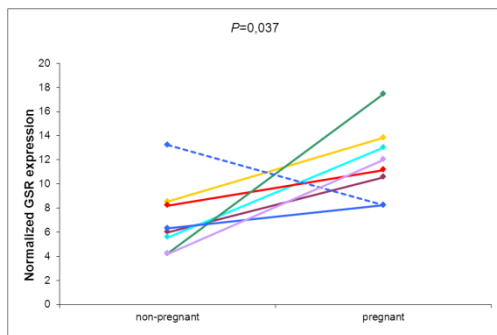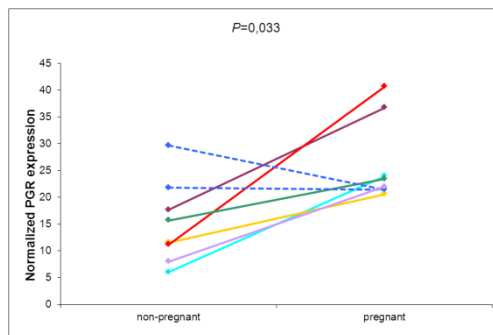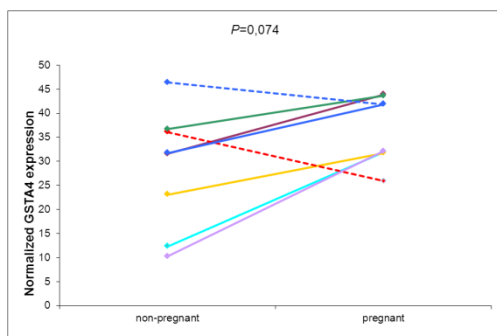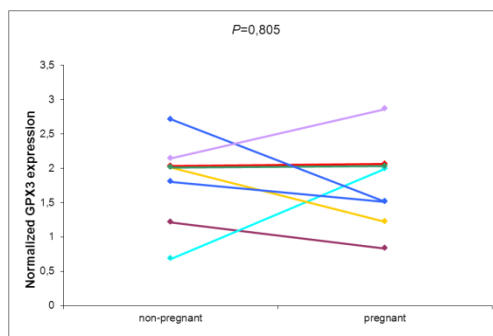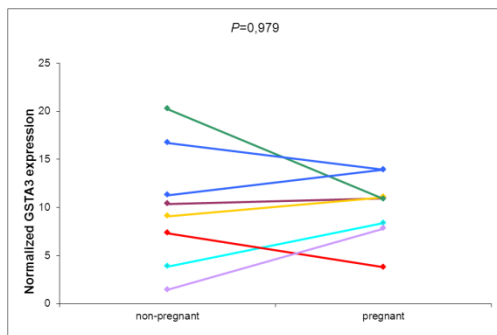

Supplement: Figure S1 — Paired t-test for the intra-patient analysis pregnant versus non-pregnant. The graphs compare for each patient the gene expression of a cumulus complex that corresponded to an oocyte that did not result in pregnancy to one that resulted in pregnancy in a subsequent single embryo frozen transfer cycle. Per patient the oocytes originate from one retrieval cycle. One patient had 2 consecutive frozen cycles, the first one not resulting in pregnancy. One color represents one patient. The dashed lines show (only in the graphs with a major trend: up or down from non-pregnant to pregnant with P<0.1) the pairs not following the major trend. Those pairs are also marked with ‘a’ in Table 7. (PDF) [file pone.0054226.s001.pdf]
