# Supplementary material for: Pregnancy Prediction in Single Embryo Transfer Cycles after ICSI Using QPCR: Validation in Oocytes from the Same Cohort
Source: PLoS One. 2013 Apr 3;8(4):e54226. doi: 10.1371/journal.pone.0054226 (PMC3616108; doi:10.1371/journal.pone.0054226)
Supplement: Table S1 — Primer sequences. (DOC) [file pone.0054226.s002.doc]

**Table S1. Primer sequences.**

| Gene Symbol | Reference Sequence Gene name | Forward and Reverse primer 5’🡪3’ |
| --- | --- | --- |
| *GPX3* | NM_002084 | ggggacaagagaagtcgaaga gccagcatactgcttgaagg |
| *GSR* | NM_000637 | caatgatcagcaccaactgc agtctttttaacctccttgacctg |
| *GSTA3* | NM_000847 | gatgccaagattgccttgat ttgtccatggctctgtaacact |
| *GSTA4* | NM_001512 | cctcaaggagagaaccctgat ggatgcatgataagcagttcc |
| *ITPR1* | NM_002222 | tacccagcggctgctaac tgcaaatcctgctcctctgt |
| *PGR* | NM_000926 | gtcatagacccccgttgcta gctaagccagcaagaaatgg |
| *SLC2A1* | NM_006516 | gtctggcatcaacgctgtc acgataccggagccaatg |
| *TGFB1* | NM_000660 | agtggttgagccgtggag gcagtgtgttatccctgctg |
| *THBS1* | NM_003246 | aatgctgtcctcgctgttg gccacagctcgtagaacagg |
